# Early discontinuation of antibiotic therapy initiated in the emergency department in older patients: a retrospective study

**DOI:** 10.1186/s12877-026-06979-w

**Published:** 2026-01-20

**Authors:** Pierre Cezard, Richard Chocron, Thomas Laurenceau, Hayat Lahjibi-Paulet, Damien Blez

**Affiliations:** 1 Service de Microbiologie, Unité Mobile d’Infectiologie, AP-HP.Centre, Hôpital Européen Georges Pompidou, Paris, 75015 France; 2 Emergency Department, AP-HP.Centre, European Georges Pompidou Hospital, Paris, 75015 France; 3 Service de Gériatrie, Hôpital Européen Georges Pompidou, AP-HP.Centre, Paris, 75015 France; 4 Faculty of Health Sciences, Sorbonne University, UFR of Medicine, Paris, 75013 France; 5 Paris Cardiovascular Research Center (PARCC), Université Paris Cité, Inserm, Paris, 75015 France; 6 Paris-Cité University, Paris, 75006 France

**Keywords:** Older, Antibiotic stewardship, Sepsis, Emergency department

## Abstract

**Background:**

Infectious diseases are a leading cause of emergency department (ED) visits in elderly patients, and their accurate diagnosis and management remain a challenge. In this context, empirical antibiotic therapy may be discontinued upon patient admission to the acute geriatric unit (AGU) during routine early clinical reassessment. However, the safety of this practice remains unclear. This study evaluated the risk of antibiotic therapy resumption due to recurrence of the initial clinical infectious syndrome after early discontinuation (≤ 48 h) in older patients admitted to the AGU.

**Methods:**

This single-center retrospective observational study included patients aged over 75 years admitted to the AGU with suspected bacterial infections who received at least one dose of antibiotic therapy in the ED.

The primary outcome was the rate of antibiotic therapy resumption for recurrent infections. The secondary outcomes included total antibiotic therapy consumption (in days), side effects, 30-day ED revisits and 30-day all-cause mortality. Noninferiority was tested with a 10% margin.

**Results:**

Among 213 patients admitted to the AGU previously receiving empirical antibiotic therapy in the ED, early antibiotic therapy discontinuation occurred in 51/213 (23.9%) patients. The “early discontinuation” group of patients had lower qSOFA scores and lactate levels. Non-inferiority for antibiotic therapy resumption due to recurrence was not demonstrated because the upper bound of the confidence interval exceeded the prespecified 10% non-inferiority margin (Absolute risk difference 4.2% 95% CI [-5.9; 14.4]). Early discontinuation had lower antibiotic therapy days (1 day *vs* 7 days, *p* < 0.001), no increase in ICU admissions (2.0% *vs* 2.5%, *p* = 1.000), and lower 30-day all-cause mortality rate (9.8% *vs* 27.2%, *p* = 0.017), but the discontinuation group was less severe at baseline (qSOFA, lactate, CRP, albumin).

**Conclusions:**

Although noninferiority was not reached in terms of antibiotic therapy resumption, early discontinuation of empirical antibiotic therapy initiated in the ED appears safe in selected older adults without sepsis criteria, with infrequent antibiotic therapy resumption and decrease in antibiotic therapy consumption with no increase in ICU transfers and mortality.

**Supplementary Information:**

The online version contains supplementary material available at 10.1186/s12877-026-06979-w.

## Background

The world population is getting older: the number of living persons aged over 80 is indeed projected to triple by 2050 [1]. Infectious diseases are a leading cause of emergency department (ED) visits in older patients [2]. Frailty is highly prevalent in this population and is strongly associated with septic shock and 30-day mortality in older ED patients with suspected infection [3]. Sepsis-related in-hospital mortality increases with age, reaching 33.9% in patients over 85 years of age [4]. Establishing a diagnosis and treating an infection in older patients are particularly challenging. Indeed, these patients often have multiple comorbidities, and the clinical manifestations of bacterial infection are frequently subtle, mild, or nonspecific and can be misleading [5, 6]. As such, fever may be absent [7], and delirium, falls, or anorexia are somettimes the only manifestations of an ongoing bacterial infection. However, these nonspecific symptoms do not accurately predict bacterial infections [8]. In this context, it is common practice to start empirical antibiotic therapy in the ED before admission to the acute geriatric unit (AGU) [9], although it could be safely put on hold while searching for signs or a diagnosis of infection [10, 11]. Once antibiotic therapy is initiated, it can be challenging for the clinical team to discontinue them, even in the absence of a confirmed infectious diagnosis [12]. This can lead to unnecessary antibiotic-related adverse events, such as antibiotic therapy toxicity, the emergence of multidrug-resistant organisms (MDROs), *Clostridioides difficile* infection, catheter-related bloodstream infections and costs for society [13, 14]. As suggested by the surviving sepsis campaign guidelines [15], it is not uncommon for this empirical antibiotic therapy to be discontinued upon admission to our hospital’s AGU based on additional medical history, negative microbiologic test results, and patient monitoring. This practice is, however, center dependent, and its safety has not been specifically addressed in elderly patients [16–18].

To investigate the safety of early antibiotic therapy discontinuation, we aimed to evaluate the risk of antibiotic therapy resumption due to recurrence of the initial infection in geriatric patients after early antibiotic therapy discontinuation.

## Methods

### Study design

We conducted a single-centre retrospective cohort study in a French tertiary care teaching hospital in Paris (Hôpital Européen Georges Pompidou, Paris, France) with 726 beds, accounting for approximately 56,000 emergency consultations yearly.

### Study population

We included all consecutive patients admitted to the hospital AGU (aged 75 years or older) after their visit to the ED and who received at least one dose of antibiotic therapy in the ED between 01/01/2022 and 12/31/2022. Patients admitted to the ED were hospitalized in the AGU, either directly or after a stay in the ED’s short-term hospitalization unit. We excluded all patients who did not ultimately receive antibiotic therapy in the ED, those for whom antibiotic therapy was initiated in the AGU, those who did not visit the ED before the AGU and those who were hospitalized outside the AGU. We also excluded patients who died or stopped antibiotic therapy for ethical reasons during the first 48 h after admission. To better characterize potential selection bias, we compared key baseline characteristics of patients included versus excluded after reviewing medical records (age, sex, ED diagnosis category and qSOFA).

### Definitions of antibiotic approaches

In the ED, empirical antibiotic therapy in older adults was initiated in the line with local protocols and international recommendations, that is, in the presence of clinical, laboratory or radiological features suggestive of bacterial infection, particularly when sepsis or shock was suspected. Early discontinuation of antibiotic therapy was defined as discontinuation within the first 48 h of antibiotic therapy initiation. In daily practice, geriatricians considered several clinical and microbiological elements when deciding to discontinue empirical antibiotic therapy, including the absence of persistent fever or localising signs of bacterial infection, the identification of an alternative non-bacterial diagnosis, negative microbiological investigations, and overall clinical stability during the first days of hospitalization. Antibiotic therapy resumption was defined as the introduction of any antibiotic therapy more than 48 h after antibiotic therapy discontinuation. We recorded the reason for antibiotic therapy resumption according to the senior attending physician of the patient (recurrence of the initial infection or healthcare-associated infection). Urinary tract infections (UTIs) included cystitis, pyelonephritis and prostatitis. Lower respiratory tract infections are divided into two entities: bacterial pneumonia (BP) and viral pneumonia (VP). Viral pneumonia with suspected bacterial superinfection was classified as BP. Thirty-day all-cause mortality was calculated from the date of ED admission, and thirty-day ED visit data were collected from the date of AGU discharge.

### Data source

Sociodemographic, administrative, and medical data were collected from the electronic medical records of each patient via DxCare® software (Dedalus). In the case of missing information, the date of the patient’s death was collected from the French public register “INSEE décès”. Patient data were collected until 30 days after hospital discharge. The ED discharge diagnosis and AGU discharge diagnosis were retrieved from the electronic medical records of each patient. Illness severity at ED presentation was approximated using the quick sequential Organ Failure Assessment (qSOFA) score, which has been proposed as a simple bedside risk-stratification tool for ED patients with suspected infection [19].

### Outcomes

The primary outcome was the rate of hospital antibiotic therapy resumption for the recurrence of the initial infection (according to the conclusion of the AGU’s senior attending physician).

The secondary outcomes were total duration of antibiotic therapy (in days), secondary transfer to the intensive care unit (ICU), antibiotic-related side effects, healthcare-associated infections (defined by their onset more than 48 h after hospital admission: catheter-related and urinary tract infections, healthcare-associated pneumonia, and *Clostridioides difficile* infection), length of hospital stay (in days), 30-day all-cause mortality rate (defined by the occurrence of a death within 30 days of the patient's hospitalization) and 30-day ED visits (defined by a new consultation in the emergency department within 30 days after the patient's discharge from the hospital). Antibiotic-related side effects were defined as new-onset acute kidney injury (AKI) according to the KDIGO criteria, allergic reactions (e.g. rash, angioedema, anaphylaxis) or neurological disorders (e.g. delirium, seizures) occurring after antibiotic therapy initiation during the index hospitalisation and considered at least possibly related to the antibiotic therapy by the AGU physician, as documented in the medical record.

### Statistical analysis

Descriptive data are presented as absolute values and percentages for categorical variables and were analysed via the χ^2^ test or Fisher’s exact test. Continuous variables are expressed as medians and interquartile ranges, and the Wilcoxon rank sum test with continuity correction was used to compare medians. The effect sizes provided for the statistical comparisons included the absolute difference (AD) and its 95% confidence interval (CI). Statistical test results were considered significant when the *p* value was < 0.05. Missing data was not imputed. For each variable, we used a complete-case approach, with the number of patients with available data reported in the tables.

We performed a noninferiority analysis to determine whether early discontinuation of antibiotic therapy within 48 h was noninferior to continued treatment. We expected that 90% of patients would not experience recurrence of the initial infection. In accordance with EMA recommendations [20], we chose a noninferiority margin of 10 percentage points. The primary analysis was based on the absolute risk difference and its two-sided 95% confidence interval. Non-inferiority was considered to be demonstrated if the upper bound of the CI for the risk difference was below the 10% margin. We also calculated the theoretical sample size that would have been required to adequately power (80% power) the primary non-inferiority hypothesis. Approximately 142 patients per group would have been required.

In an exploratory analysis, we fitted a multivariable logistic regression model for 30-day all-cause mortality. The model included group (early discontinuation vs continuation of antibiotic therapy), age, sex, qSOFA ≥ 2, lactate level on admission, and ED pneumonia diagnosis as covariate. To limit overfitting given the small number of deaths, we used a complete-case approach and restricted the number of covariates a priori. Results are presented as adjusted odds ratios (aORs) with 95% confidence intervals (CIs). For the primary outcome of antibiotic therapy resumption due to recurrence of the initial infection, the main analysis remained unadjusted. Because of the limited number of recurrence events, we did not construct a multivariable model for this endpoint and interpreted the results with caution.

Statistical analysis was performed via R studio software, including R version 4.3.1 (R Foundation for Statistical Computing, Vienna, Austria).

### Ethics approval and consent to participate

This study was approved by the ethics committee of AP-HP (CERAPHP.5) (IRB registration: #00011928). The collection and storage of data followed the French General Data Protection Regulations (GDPR) and this study adhered to the 1964 Declaration of Helsinki and its later amendments. Because this was a retrospective analysis of de-identified routinely collected data that posed no more than minimal risk, the IRB waived the requirement for written informed consent, in accordance with French public-health legislation (*Code de la Santé Publique*, *Articles L1122-1 à L1122-2*). Written information was sent by mail to every patient alive at the time of inclusion.

## Results

**Fig. 1:**
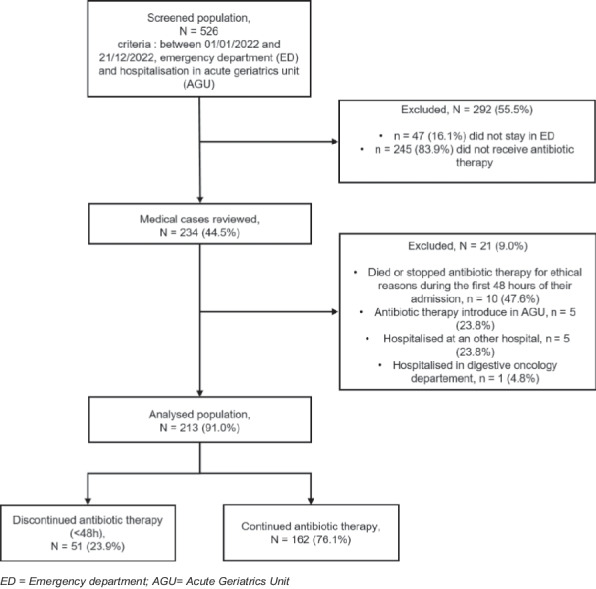
Flow diagram of the study. *ED* = *Emergency department; AGU* = *Acute Geriatrics Unit*

Among 526 patients admitted to the AGU after the ED visit, 234 received at least one dose of antibiotic therapy, and 213 patients were included in the analysis (Fig. 1), including 51/213 (23.9%) with early discontinuation of antibiotic therapy. Baseline characteristics are presented in Table 1. Regarding the baseline characteristics of the 21 patients excluded after review of medical records, 47.6% were women, the median age was 90 years (IQR 87–95), on arrival at the ED 76.1% had a qSOFA score between 0 and 1, and the most frequent primary diagnosis in the ED was pneumonia (47.6%). Regarding the early discontinuation antibiotic therapy group and the continued antibiotic therapy group, the median activities of daily living and instrumental activities of daily living scales were 4.0 (1.0–5.0) and 1.0 (0.0–4.0), respectively. The most common admission diagnoses at ED discharge were pneumonia (123/213, 57.7%) and UTIs (75/213, 35.2%). The antibiotic therapy used and the pathogens considered to be responsible for the initial infections are shown in Tables 2 and 3, respectively.

**Table 1:** Baseline participant characteristics

Characteristic	All (*n* = 213)	Discontinued ATB (*n* = 51)	Continued ATB (*n* = 162)	*p* value
Age, median (IQR), y	90 (86–94)	91 (87–95)	90 (86–94)	0.816
Female, No (%)	126 (59.2)	33 (64.7)	93 (57.4)	0.446
Modified Charlson Comorbidity Index, No. (%)				
0–2	88 (41.3)	24 (47.1)	64 (39.5)	0.534
3–4	63 (29.6)	15 (29.4)	48 (29.6)	
≥ 5	62 (29.1)	12 (23.5)	50 (30.9)	
Nursing home, No. (%)	38 (17.8)	5 (9.8)	33 (20.4)	0.131
Antibiotic within 3 months, No. (%)	40 (18.8)	9 (17.6)	31 (19.1)	0.975
Hospitalized within 3 months, No. (%)	44 (20.7)	10 (19.6)	34 (21.0)	0.989
Time of admission, No. (%)				
Night	85 (39.9)	23 (45.1)	62 (38.3)	0.481
qSOFA, No. (%)				
0–1	176 (82.6)	50 (98.0)	126 (77.8)	< 0.001
Time to onset of symptoms, median (IQR), d	1 (1–3)	1 (1–2)	1 (1–3)	0.169
Diagnoses upon discharge from ED				
UTI, No. (%)	75 (35.2)	17 (33.3)	58 (35.8)	0.867
Pneumonia, No. (%)	123 (57.7)	29 (56.9)	94 (58.0)	1.000
Skin and Soft Tissue infection, No. (%)	12 (5.6)	3 (5.9)	9 (5.6)	1.000
Biliary tract infection, No. (%)	3 (1.4)	1 (2.0)	2 (1.2)	0.562
Digestive infection, No. (%)	7 (3.3)	0 (0.0)	7 (4.3)	0.201
Other infection, No. (%)	5 (2.3)	1 (2.0)	4 (2.5)	1.000
Unknown, No. (%)	5 (2.3)	2 (3.9)	3 (1.9)	0.595
No infection, No. (%)	1 (0.5)	0 (0.0)	1 (0.6)*	1.000

Data are presented as n (%) or median [IQR]. Percentages are calculated using the number of patients with available data as the denominator. Missing data were as follows: ADL, 10/213 patients (4.7%); IADL, 20/213 (9.4%); Time to onset of symptoms, 77/213 (36.2%). All other variables in this table had complete data. The Wilcoxon rank sum test with continuity correction was used to compare medians, and the χ^2^ test or Fisher’s exact test was used to compare proportions. Time of admission (Day = 8:30am-6:29 pm/Night = 6:30 pm-8:29am)

*ATB* Antibiotic therapy, *IQR* Interquartile range, *ED* Emergency department, *UTI* Urinary tract infection, *qSOFA* Quick SOFA score, which includes 1 point for each of the 3 criteria: respiratory rate ≥ 22 breaths/min, systolic blood pressure ≤ 100 mmHg, and altered mental status. A score ≥ 2 suggests sepsis

^*^Preventive antibiotic therapy for a facial fracture, continued for 3 days

**Table 2:** Antibiotic therapy used in the ED and AGU

ED, No. (%)	Overall (*n* = 213)	AGU, No. (%)	Discontinued ATB (*n* = 51)	Continued ATB (*n* = 162)
Amikacine	15 (7.0)	Amikacine	0 (0.0)	2 (1.2)
Amoxicilline	10 (4.7)	Amoxicilline	3 (5.9)	26 (16.0)
Augmentin	70 (32.9)	Augmentin	3 (5.9)	61 (37.7)
Azythromycine	1 (0.5)	Azythromycine	0 (0.0)	1 (0.6)
		Cefazoline*	0 (0.0)	1 (0.6)
Cefepime	1 (0.5)	Cefepime	1 (2.0)	2 (1.2)
Cefotaxime	132 (62.0)	Cefotaxime	2 (3.9)	67 (41.4)
		Ceftazidime*	0 (0.0)	2 (1.2)
Ceftriaxone	4 (1.9)	Ceftriaxone	0 (0.0)	4 (2.5)
Ciprofloxacine	1 (0.5)	Ciprofloxacine	0 (0.0)	3 (1.9)
Clindamycine	2 (0.9)	Clindamycine	0 (0.0)	1 (0.6)
Levofloxacine	4 (1.9)	Levofloxacine	0 (0.0)	15 (9.3)
Meropenem	1 (0.5)	Meropenem	0 (0.0)	3 (1.9)
Metronidazole	22 (10.3)	Metronidazole	0 (0.0)	25 (15.4)
Piperacilline-Tazobactam	8 (3.8)	Piperacilline-Tazobactam	1 (2.0)	23 (14.2)
Spiramycine	26 (12.2)	Spiramycine	0 (0.0)	1 (0.6)
		Sulfamethoxazole-trimethoprime*	1 (2.0)	5 (3.1)
		Teicoplanine*	0 (0.0)	2 (1.2)
		Vancomycine*	0 (0.0)	1 (0.6)

^*^Newly introduced in Acute Geriatric Unit (AGU). Some patients received several molecules of probabilistic antibiotic therapy (ATB) in the emergency department (ED)

**Table 3:** Pathogens responsible for the infectious syndrome

Microbiological diagnosis No. (%)	Overall (*n* = 213)
Absence of microbiological diagnosis	142 (66.7)
*Enterobacterales*	51 (23.9)
*Pseudomonas sp.*	5 (2.3)
*Streptococcus sp.*	13 (6.1)
*Enterococcus sp.*	4 (1.9)
Others Bacteria	
*Acinetobacter* sp.	1 (0.5)
*Bacteroides* sp.	1 (0.5)
*Haemophilius influenzae*	1 (0.5)
*Legionella pneumophila*	1 (0.5)
*Stenotrophomonas maltophilia*	1 (0.5)
Viruses	
Adenovirus	1 (0.5)
Enterovirus	6 (2.8)
Influenza A virus	7 (3.3)
Human metapneumovirus	1 (0.5)
Rhinovirus	6 (2.8)
SARS-CoV-2	34 (16.0)
Respiratory Syncytial Virus	6 (2.8)

Percentages are calculated among patients with available data. Microbiological variables were only recorded when the corresponding test was performed and/or positive, which explains the high proportion of missing values for detailed organism. In this table, missing values therefore mostly reflect tests that were not performed or cultures with no growth, rather than missing documentation. Enterobacterales included (E. Coli, *n* = 37; K. oxytoca, *n* = 2; K. pneumoniae, *n* = 6; E. cloacae, *n* = 1; P. mirabilis, *n* = 4; P. vulgaris, *n* = 1). Pseudomonas sp. included (P. aeruginosa, *n* = 4;, P. oryzabitans, *n* = 1). Stretococcus sp. included (S. pneumoniae, *n* = 10; S. mitis, *n* = 1; S. gorgonii, *n* = 1; S. anginosus, *n* = 1). Enterococcus sp. included (E. faecalis, *n* = 3; E. faecium, *n* = 1)

Early discontinuation of antibiotic therapy occurred in 29/123 patients (23.6%) with suspected pneumonia and in 17/75 patients (22.7%) with suspected UTI. The two reasons for antibiotic therapy discontinuation were the absence of infection (37/51, 72.5%) and the diagnosis of viral infection (14/51, 27.5%). Early discontinuation group patients had a less severe initial presentation, on the basis of their qSOFA score (qSOFA ≥ 2: 2% *vs* 22.2%, *p* < 0.001) and lactate levels (1.5 *vs* 1.9 mmol/L, *p* = 0.012); had lower inflammatory markers (leukocytes 10.2 *vs* 12.6 G/L, *p* = 0.002; C-reactive protein (CRP) 38 *vs* 86 mg/L, *p* < 0.001); and had higher albumin levels (35 *vs* 31 g/L, *p* < 0.001). The biological parameters at ED admission are presented in Table 4.

**Table 4:** Biological parameters on ED admission

Biological parameters	Overall (*n* = 213)	Discontinued ATB (*n* = 51)	Continued ATB (*n* = 162)	*p* value
Temperature, median (IQR), °C	37 (36.5–37.8)	37 (36.5–37.5)	37 (36.5–37.9)	0.143
Leukocyte count, median (IQR), G/L	11.8 (8.8–16.1)	10.2 (7.9–12.3)	12.6 (9.3–17.2)	0.002
Hemoglobin level, median (IQR), g/dL	12.3 (10.9–13.5)	12.5 (11.2–13.4)	12.3 (10.8–13.5)	0.575
Platelet count, median (IQR), G/L	236 (183–318)	206 (169–286)	242 (192–333)	0.038
Serum creatinine, median (IQR), µmol/L	99 (72–140)	90 (67–119)	102 (74–147)	0.074
Albumin level, median (IQR), g/L	32 (29–35)	35 (31–38.5)	31 (29–34)	< 0.001
CRP, median (IQR), mg/L	75 (29–141)	38 (14–88)	86 (34–149)	< 0.001
Lactate level, median (IQR), mmol/L	1.8 (1.3–2.6)	1.5 (1.2–1.8)	1.9 (1.4–2.8)	0.012

Data are presented as median [IQR]. Percentages are calculated using the number of patients with available data as the denominator. Missing data were as follows: Platelet count, 4/213 (1.9%); C-reactive protein, 2/213 (0.9%); lactate level, 95/213 (44.6%); albumin level, 29/213 (13.6%); Serum creatinine, 3/213 (1.4%); All other variables in this table had complete data

*ED* Emergency Department, *ATB* Antibiotic therapy, *IQR* Interquartile range, *CRP* C reactive protein

Differences between ED diagnoses and AGU diagnoses at discharge are shown in Table 5. Overall, 64/213 (30.0%) patients had a final diagnosis between ED and AGU discharge, and 54/213 (25.4%) of the empirically treated patients in the ED did not have a final diagnosis of bacterial infection (39/213 (18.3%) had no infection, and 15/213 (7.0%) had viral infection). The discharge diagnoses from the AGU are presented in Table 6. Six patients in the continued antibiotic therapy group received antibiotic therapy for more than 48 h despite a final diagnosis of “no infection” due to a delayed evaluation (e.g., antibiotic therapy was interrupted at 72 h).

**Table 5:** Number of corrected diagnostics

Diagnoses	Overall in ED (*n* = 213)	Overall in AGU (*n* = 213)	Corrected diagnostics
UTI, No. (%)	75 (35.2)	48 (22.5)	27 (36.0)
Pneumonia, No. (%)	123 (57.7)	91 (42.7)	32 (26.0)
Skin and Soft Tissue infection, No. (%)	12 (5.6)	8 (3.8)	4 (33.0)
Biliary tract infection, No. (%)	3 (1.4)	2 (0.9)	1 (33.0)
Digestive infection, No. (%)	7 (3.3)	7 (3.3)	0 (0.0)

*ED* Emergency Department, *AGU* Acute Geriatric Unit, *UTI* Urinary Tract Infection

**Table 6:** Discharge diagnosis from the AGU

AGU	Overall (*n* = 213)	Discontinued ATB (*n* = 51)	Continued ATB (*n* = 162)	*p* value
UTI, No. (%)	48 (22.5)	1 (2.0)	47 (29.0)	< 0.001
Pneumonia, No. (%)	91 (42.7)	3 (5.9)	88 (54.3)	< 0.001
Skin and Soft Tissue infection, No. (%)	8 (3.8)	0 (0.0)	8 (4.9)	0.203
Biliary tract infection, No. (%)	2 (0.9)	0 (0.0)	2 (1.2)	1.000
Digestive infection, No. (%)	7 (3.3)	0 (0.0)	7 (4.3)	0.201
Other infection, No. (%)	12 (5.6)	0 (0.0)	12 (7.4)	0.074
Viral infection, No. (%)	15 (7.0)	14 (27.5)	1 (0.6)	< 0.001
Unknown, No. (%)	0 (0.0)	0 (0.0)	0 (0.0)	
No infection, No. (%)	39 (18.3)	33 (64.7)	6 (3.7)	< 0.001

*AGU* Acute Geriatric Unit, *ATB* Antibiotic therapy, *UTI* Urinary Tract Infection

### Primary outcome

Antibiotic therapy resumption for recurrence of the initial infection occurred in 5/51 (9.8%) patients in the early discontinuation group and 9/162 (5.6%) in the continued antibiotic therapy group (Table 7), corresponding to an absolute risk difference of 4.2 percentage points (95% CI −5.9 to 14.4). Non-inferiority was not demonstrated, as the upper bound of the confidence interval exceeded the prespecified 10% non-inferiority margin.

**Table 7:** Primary and secondary outcomes

Outcomes	Discontinued ATB (*n* = 51)	Continued ATB (*n* = 162)	AD – IC95% or *p*-value
Primary outcomes			
Antibiotic therapy resumption for recurrence of the initial infection, No. (%)	5 (9.8)	9 (5.6)	4.2 [−5.9; 14.4]
Antibiotic therapy resumption all cause, No. (%)	8 (15.7)	13 (8.0)	7.7 [−4.4; 19.8]
Antibiotic therapy resumption for healthcare-associated infection*, No. (%)	3 (5.9)	4 (2.5)	3.4 [−4.8; 11.6]
Secondary outcomes			
Total duration of antibiotic therapy, median (IQR), d	1 (1–2)	7 (5–11)	< 0.001
Length of hospital stay, median (IQR), d	11(8–14)	10.5 (7–14.8)	0.570
Healthcare-associated infections, No. (%)	9 (17.6)	26 (16.0)	1.6 [−11.6; 14.8]
Postadmission acute kidney injury, No. (%)	8 (15.7)	31 (19.3)	−3.6 [−16.4; 9.5]
Admission to ICU, No. (%)	1 (2.0)	4 (2.5)	−0.5 [−5.5; 4.5]
30-day ED visits, No. (%)	6 (11.8)	12 (7.4)	4.4 [−6.7; 15.4]
30-day all-cause mortality, No. (%)	5 (9.8)	44 (27.2)	−17.4 [−29.3; −5.4]

*AD* Absolute difference in proportion with a 95% confidence interval, *ATB* Antibiotic therapy, *ICU* Intensive care unit, *ED* Emergency Department

^*^Healthcare-associated infection: catheter-related infection (*n* = 1 in the discontinued group), Healthcare-associated pneumonia (*n* = 3 in the continued group and discontinued group), UTI = urinary tract infection (*n* = 2 in the continued group). Some patients have had multiple healthcare-associated infections. The Wilcoxon rank sum test with continuity correction was used to compare medians

Because of the small number of recurrence events, no multivariable model was fitted for antibiotic therapy resumption. The analysis was therefore limited to unadjusted comparisons of proportions and risk differences, and the results should be interpreted with caution.

The reasons for antibiotic therapy resumption are detailed in Table S1. Among the five patients in the early discontinuation group who resumed antibiotic therapy, one patient returned to the ED after AGU discharge for non-infectious reasons, and none of them died or required ICU admission. Healthcare-associated infections occurred in 7/213 (3.3%) patients, including 3/51 (5.9%) in the early discontinuation group (healthcare-associated pneumonia (*n* = 3) and catheter-related infection (*n* = 1), and one patient had both).

### Secondary outcomes

Patients in the early discontinuation group had lower antibiotic therapy days (1 *vs* 7 days; *r* = 0.61, *p* < 0.001). There was no significant increase in secondary ICU admissions or acute kidney injury in the early discontinuation group, and the 30-day all-cause mortality rate was lower (Table 7). Although the rates of healthcare-associated infections were similar between early discontinuation and other methods (17.6% *vs* 16.0%), we were unable to demonstrate noninferiority, with a 10% margin for this endpoint. Two *Clostridioides difficile* infections occurred in the continued antibiotic therapy group.

### Adjusted analysis of 30-day all-cause mortality

In the exploratory multivariable logistic regression model including group, age, sex, qSOFA ≥ 2, lactate level, and ED pneumonia diagnosis (complete-case *n* = 118), early discontinuation of antibiotic therapy was not associated with higher odds of 30-day all-cause mortality. The adjusted odds ratio (aOR) for 30-day all-cause mortality in the early discontinuation group compared with the continuation group was 0.34 (95% CI 0.07—1.13; *p* = 0.11). Age, sex, qSOFA ≥ 2 and ED pneumonia diagnosis were not independently associated with 30-day mortality in this model. Higher lactate levels were positively associated with 30-day all-cause mortality (aOR 1.27 per 1 mmol/L increase, 95% CI 1.00—1.64; *p* = 0.05), with a borderline statistical significance.

## Discussion

In this single-center retrospective study, early discontinuation of empirical antibiotic therapy occurred in 23.9% (51/213) of elderly patients admitted to the AGU after an ED visit. The rate of antibiotic therapy resumption was low (< 10%) in this group; however, noninferiority was not reached.

Notably, the early discontinuation group had significantly lower antibiotic therapy consumption with no increase in ICU admissions or 30-day all-cause mortality. Moreover, none of the five patients who required antibiotic therapy resumption experienced infection-associated complications.

This is the first study to assess real-life geriatric patients (median age of 90 years) who received empirical antibiotic therapy in the ED. After early discontinuation, antibiotic therapy resumption during hospitalization for recurrence of the initial infection was infrequent (< 10%) and did not lead to any serious adverse outcomes. These findings are consistent with those of another retrospective study, which showed that discontinuation within 24 h as part of an antibiotic therapy stewardship program reduced the total treatment duration without increasing 14-day mortality or infection-related readmissions [17]. However, this study did not focus on elderly patients.

We selected a 48-h cut-off for early discontinuation, as it reflects the typical time frame between antibiotic therapy initiation in the ED and admission to the AGU. While some infections may resolve with treatment courses as short as three days, a duration of two days is usually considered inadequate for most bacterial infections [21]. Therefore, the absence of recurrence is a reasonable indicator of the absence of bacterial infection.

In our setting, empirical antibiotic therapy in the ED is broadly aligned with international recommendations for patients with suspected serious infection. In older adults, antibiotics are usually initiated when clinical, laboratory or radiological features are suggestive of bacterial infection, particularly when sepsis or septic shock is suspected, with priority given to early treatment in higher-risk situations. Our findings nevertheless show that almost one quarter of older patients who received empirical antibiotic therapy in the ED ultimately had their antibiotic therapy discontinued after geriatric reassessment and did not have a final diagnosis of bacterial infection. This highlights a substantial opportunity to strengthen antibiotic stewardship, as well as to systematically reassess the need for continued therapy throughout hospitalization.

The most common diagnoses are pneumonia and UTIs, which are particularly challenging in elderly patients because of nonspecific symptoms and multiple possible causes of oxygen requirements [6]. This was illustrated by the 29.8% rate of observed discordance between the ED’s diagnosis and the final AGU in our study. The reassessment after 48 h allows for consolidating the clinical hypotheses based on early microbiological and imaging results. The mortality rate in our study was slightly lower than the 30% reported in the literature [22, 23] and was even lower in the early discontinuation group. The lower mortality observed in the discontinuation group is consistent with their lower baseline severity. All direct comparisons of mortality between the two groups should be interpreted with caution given this strong confounding factor.

Healthcare-associated adverse events, such as nosocomial infections and acute kidney injury, were also relatively frequent in this population. While not all these events can be directly attributed to antibiotic therapy use, elderly patients are known to be at increased risk for adverse events related to antibiotic therapy [24–26]. Therefore, their rational use should contribute to improving the overall outcome of these patients.

Our study has several limitations. In this single-center retrospective study, the small sample size and limited statistical power hindered our ability to demonstrate noninferiority and to draw firm conclusions regarding the primary outcome.

The retrospective design also carries the risk of missing data. Several key variables had substantial missingness, particularly duration of symptoms, lactate level and some biological markers. We used a complete-case approach without imputation, which reduced the effective sample size for some analysis and may have introduce selection bias if data were not missing at random. However, for most variables data were available for the entire cohort, and when missing values were present, they accounted for less than 10% of observations.

Moreover, patients without early discontinuation more frequently had confirmed bacterial infections and severe initial presentations, which likely influenced the outcomes. Also, we used qSOFA as a proxy for baseline illness severity. However, qSOFA score has shown limited sensitivity and only moderate prognostic performance for sepsis and mortality in ED populations [19]. Residual confounding by illness severity is therefore likely, even after including qSOFA in our adjusted analyses.

The low number of events also prevented adjustment for potential confounders. However, in an exploratory adjusted analysis, early discontinuation of antibiotic therapy was not associated with an increased risk of 30-day mortality after controlling for age, sex, qSOFA ≥ 2, lactate level and ED pneumonia diagnosis. Although the confidence interval was wide and compatible with both a substantial reduction and a modest increase in mortality, these findings are reassuring and suggest that early discontinuation may be safe in carefully selected patients without sepsis criteria. Given the observational design, the complete-case approach, and the small number of deaths, this adjusted analysis should be interpreted with caution, and causal effects cannot be inferred.

Finally, one important limitation is that our protocol did not include systematic antibiotic-stewardship team consultation, nor was the intervention structured as a standardized “antibiotic therapy review”. Consequently, the description of our intervention may lack sufficient detail for straightforward reproducibility, and the findings should be interpreted with caution when extrapolating to stewardship programs in other settings. Future studies should incorporate stewardship expertise and employ a rigorously defined antibiotic therapy review framework to increase generalizability.

Despite these biases, the overall low failure rate of antibiotic therapy discontinuation supports the relative safety of this approach.

## Conclusions

In conclusion, we did not demonstrate non-inferiority regarding antibiotic therapy resumption. Nonetheless, early discontinuation of empirical antibiotic therapy appeared safe in selected older adults without sepsis criteria, with a low rate of antibiotic therapy resumption, a reduction in overall antibiotic therapy uses and no increase in ICU transfer or mortality. Larger and prospective studies are needed to confirm these findings and support wider implementation.

## Supplementary Information

Supplementary Material 1.

## Data Availability

The data presented in this study are available upon request from the corresponding author due to privacy restrictions.
