# Supplementary material for: Early discontinuation of antibiotic therapy initiated in the emergency department in older patients: a retrospective study
Source: BMC Geriatr. 2026 Jan 20;26:168. doi: 10.1186/s12877-026-06979-w (PMC12879421; doi:10.1186/s12877-026-06979-w)
Supplement: Supplementary file 1 — Supplementary Material 1. [file 12877_2026_6979_MOESM1_ESM.pdf]

**Supplementary Appendix**

*Supplement to: Cezard P, Chocron R, Laurenceau T, Lahjibi-Paulet H, Blez D*
*Early discontinuation of antibiotic therapy initiated in the emergency department in older patients: a retrospective study. This appendix has been provided by the authors to give readers additional information about the work.*

**Table S6. Synthesized description of clinical cases of antibiotic therapy resumption for recurrence of the initial clinical syndrome in the early antibiotic therapy discontinuation group.**

| Patient | History of the disease |
|---|---|
| N°1 | 95-year-old man arrives at night with febrile cough evolving for 24 hours. Charlson Comorbidity Index 0, autonomous in daily activities. On admission, apyretic at 37°C, qSOFA at 0, biological inflammatory syndrome with 11 G/L leukocytes and CRP at 160 mg/L, chest X-ray showing a focus of parenchymal condensation. Triplex nasopharyngeal PCR (COVID, influenza, RSV) negative, antigenuria legionella and pneumococcus negative. Diagnosis of pneumonia on discharge from emergency department, with initiation of antibiotic therapy with CEFOTAXIME. Early discontinuation due to viral pulmonary infection, with a second positive nasopharyngeal PCR for SARS CoV 2 on arrival in geriatrics. Three days after discontinuation, recurrence of biological inflammatory syndrome without fever or increased oxygen requirements. Chest CT scan showed infectious pneumonia, requiring reintroduction of CEFOTAXIME. Favourable evolution afterwards, diagnosis of undocumented bacterial pneumonia on discharge from geriatric care. |
| N°2 | 97-year-old man arrives home at night with a fall, fever and need for oxygen therapy with nasal cannula, Charlson Comorbidity Index 2, autonomous in acts of daily living. On admission, fever 38.7°C, qSOFA = 1, leukocytes 6.7 G/L and CRP slightly increased to 31.6 mg/L, chest X-ray inconclusive, PCR SARS CoV 2 positive, antigenuria legionella and pneumococcus negative. Emergency diagnosis: superinfected SARS CoV 2 pneumonia. On arrival in the geriatric ward, antibiotic therapy discontinued, as there was no longer any evidence of a nonsuperinfected viral pulmonary infection. Evolution marked by a paucisymptomatic SARS CoV 2 infection, but increased cough and sputum without fever and no increase in the biological inflammatory syndrome 6 days after discontinuation. Reintroduction of antibiotic therapy in the face of a positive ECBC for *enterobacterales*. Discharge diagnosis: Bronchitis and SARS CoV 2 respiratory infection. |
| N°3 | 87-year-old man, arriving at night with cough and dyspnea evolving for 2 days. Charlson Comorbidity Index 6, autonomous in daily activities. On admission, apyretic at 37°C, qSOFA = 1, leukocytes 9 G/L and CRP slightly elevated at 37 mg/L, chest X-ray finding a focus of parenchymal condensation, triplex PCR negative, antigenuria negative, ECBC finding *Candida albicans*. Discharge diagnosis: Pneumonia probably bacterial. On arrival in the geriatric ward, antibiotic therapy was discontinued as there was no clinical or radiological evidence of pneumonia after reassessment of the patient. A chest CT scan was performed, but found no evidence of pneumonia. Three days after discontinuation, cough and sputum increased, as did the biological inflammatory syndrome. Antibiotic therapy with AMOXICILLIN was reintroduced for 5 days. Favourable outcome on discharge from geriatric care: Exacerbation of COPD. |

| | |
|---|---|
| N°4 | 94-year-old man arrives at night with febrile dyspnea, shivering and need for oxygen therapy, 5 days of progression. Charlson Comorbidity Index 8, autonomous in acts of daily living. On admission, fever at 37.7°C, qSOFA at 1, absence of biological inflammatory syndrome with leukocytes at 3.2 G/L and CRP at 4.9 mg/L, chest X-ray showing a parenchymal focus, positive SARS CoV 2 PCR, ECBC finding oro-pharyngeal flora and negative antigenuria. Discharge diagnosis was superinfected SARS CoV 2 pneumonia. Antibiotic therapy was discontinued on arrival in the geriatric ward, as the geriatrician diagnosed a picture suggestive of SARS CoV 2 viral infection, without superinfection. Three days after discontinuation, persistent cough with dirty sputum and recurrence of fever. New ECBC positive for *Pseudomonas aeruginosa*, leading to resumption of antibiotic therapy. Discharge diagnosis: SARS CoV 2 pneumonia superinfected with *Pseudomonas aeruginosa*. |
| N°5 | 82-year-old woman, arriving at night with a fall at home, prolonged standing on the floor and fever evolving for 24 hours. Charlson Comorbidity Index 1, autonomous in daily activities. On arrival, temperature 38.1°C, qSOFA 0, moderate biological inflammatory syndrome with 11.8 G/L leukocytes and 16.4 mg/L CRP, chest X-ray showing no focus, SARS CoV 2 PCR positive, ECBU finding 20.10^3/mm3 leukocytes, gram-negative bacillus on direct examination, *Escherichia coli* culture positive at 10^6. Discharge diagnosis: urinary tract infection associated with SARS CoV 2 respiratory infection. Antibiotic therapy discontinued on arrival in geriatric ward in the absence of functional urinary tract signs and in the presence of SARS CoV 2 respiratory infection, but fever recurs and functional urinary tract signs appear 24 hours after discontinuation. Geriatric discharge diagnosis: urinary tract infection and SARS CoV 2 respiratory infection. |
